# Genomic analysis uncovers novel candidate genes related to adaptation to tropical climates and milk production traits in native goats

**DOI:** 10.1186/s12864-024-10387-y

**Published:** 2024-05-14

**Authors:** Chenxi Zhang, Hojjat Asadollahpour Nanaei, Niloufar Jafarpour Negari, Mahmoud Amiri Roudbar, Zeinab Amiri Ghanatsaman, Zhannur Niyazbekova, Xiaojun Yang

**Affiliations:** 1 College of Animal Science and Technology, Northwest A&F University, Yangling, Shaanxi 712100 China; 2 College of Life Sciences, Northwest A&F University, Yangling, 712100 China; 3 Animal Science Research Department, Fars Agricultural and Natural Resources Research and Education Center, Agricultural Research, Education and Extension Organization (AREEO), Shiraz, Iran; 4 Department of Animal Science, Shahid Bahonar University of Kerman, Kerman, 7616914111 Iran; 5 Department of Animal Science, Safiabad-Dezful Agricultural and Natural Resources Research and Education Center, Agricultural Research, Education and Extension Organization (AREEO), Dezful 333, Iran; 6 Key Laboratory of Animal Genetics, Breeding and Reproduction of Shaanxi Province, College of Animal Science and Technology, Northwest A&F University, Yangling, Shaanxi 712100 China

**Keywords:** Population genomics, Pakistan indigenous goats, Whole-genome sequence, Adaptation, Candidate genes

## Abstract

**Background:**

Since domestication, both evolutionary forces and human selection have played crucial roles in producing adaptive and economic traits, resulting in animal breeds that have been selected for specific climates and different breeding goals. Pakistani goat breeds have acquired genomic adaptations to their native climate conditions, such as tropical and hot climates. In this study, using next-generation sequencing data, we aimed to assess the signatures of positive selection in three native Pakistani goats, known as milk production breeds, that have been well adapted to their local climate.

**Results:**

To explore the genomic relationship between studied goat populations and their population structure, whole genome sequence data from native goat populations in Pakistan (*n* = 26) was merged with available worldwide goat genomic data (*n* = 184), resulting in a total dataset of 210 individuals. The results showed a high genetic correlation between Pakistani goats and samples from North-East Asia. Across all populations analyzed, a higher linkage disequilibrium (LD) level (– 0.59) was found in the Pakistani goat group at a genomic distance of 1 Kb. Our findings from admixture analysis (*K* = 5 and *K* = 6) showed no evidence of shared genomic ancestry between Pakistani goats and other goat populations from Asia. The results from genomic selection analysis revealed several candidate genes related to adaptation to tropical/hot climates (such as; *KITLG*, *HSPB9*, *HSP70*, *HSPA12B*, and *HSPA12B*) and milk production related-traits (such as *IGFBP3*, *LPL*, *LEPR*, *TSHR*, and *ACACA*) in Pakistani native goat breeds.

**Conclusions:**

The results from this study shed light on the structural variation in the DNA of the three native Pakistani goat breeds. Several candidate genes were discovered for adaptation to tropical/hot climates, immune responses, and milk production traits. The identified genes could be exploited in goat breeding programs to select efficient breeds for tropical/hot climate regions.

**Supplementary Information:**

The online version contains supplementary material available at 10.1186/s12864-024-10387-y.

## Background

For thousands of years, domesticated animals have played important roles in human society and have been genetically adapted to a variety of environmental circumstances [1–3]. They have occupied a wide range of roles including; source of energy, food, material for construction, herding, and manure for primarily rural farmers. Based on recent paleogenomic evidence, goat (*Capra hircus*) were one of the first animals domesticated for use as livestock some 10,000 years ago from multiple wild bezoars in Southwest Asia [2]. Since domestication, they have spread throughout the globe, and currently there are more than 1 billion farmed goats with over 210 recognized breeds [4–6].

Generally, indigenous goat breeds have excellent adaptability to local climates, such as tropical and arid environments [7]. They have a high level of vitality, resistance to endemic diseases and low input regimes [8]. Today, local goats have become an important supply of high-quality products in the developing world through the conversion of feed from noncompetitive sources. Due to their ability to survive in harsh climates, they have increasingly become a profitable business for farmers in local regions [4, 9]. According to the FAO, more than 90% of goat populations are inhabited in developing countries (https://www.fao.org/faostat/en/#data/QCL), and Pakistan with more than 53 million heads and around 25 breeds is ranked third after China and India [10, 11]. Thus, goat farming is an important part of the livestock sector in this country. Among the local Pakistani goats, some of them are popular for their higher levels of milk production traits. For example, Beetal goat is a well-known breed used for dairy products in Pakistan due to its high production capacity (average 1.2-liters milk per day) and also its beauty [12]. Damani is another local dairy goat breed that originated in the Bannu and Dera Ismail Khan districts in NWF Province, Pakistan. Kamori goat, which is mainly raised for milk production (average 1.5-2-liters of milk per day), is popularly known as the cow of the poor farmer in the country [13, 14].

In the last ten years, numerous genetic studies have been carried out in order to explore genetic diversity and identify loci related to climate change and production traits in different goat breeds [15–18]. However, there are only a few genomic studies focusing on Pakistani native goats, and the majority of these studies have been carried out using limited microsatellite marker resources, which prevents them from detecting novel variation at the complete sequence level [19, 20].

Here we explored a thorough genetic analysis of worldwide goat genomes (*n* = 210), including seven populations from different geographical regions, to investigate population genetic diversity, genetic structure, and also the signatures of selection analysis. Our findings from selective genomic analysis revealed new genomic footprints related to adaptation to local climates and milk production traits that may be under natural/artificial selection in local goat populations.

## Methods

### Quality control checking, read mapping and SNP calling

The experimental samples were collected from the Sindh Agriculture University, Tandojam, Pakistan. The studied animals were not anesthetized or euthanized in order to perform this study. No goat individuals died in this study, and all animals stayed healthy after collecting blood samples. About 15 milliliters of whole blood (*n* = 26) were collected from each native goat. By using the standard phenol-chloroform extraction method, DNA was extracted from blood samples. Sequence data were generated using the Illumina Hiseq 2500. In addition, previously published genome data from worldwide domestic goat samples (*n* = 184) was collected from the Sequence Read Archive (https://trace.ncbi.nlm.nih.gov/Traces/sra). Supplementary Table S1 provides details on the samples utilized in this study. By applying the FASTQC program (Version 0.4.2), quality control checks on raw high throughput sequence data were carried out (https://www.bioinformatics.babraham.ac.uk/projects/fastqc/), and low quality base pairs and adapters were then filtered from the original paired-end sequences by Trimmomatic software (version 0.38.1) [21]. BWA (mem algorithm) (Version 0.7.15) [22] was performed to map all pre-processed reads to the reference genome (GCF_001704415.1, ARS1). The SAMtools program was applied for manipulating SAM (.sam) files and converting files between different formats [23]. In order to minimize false positives, we removed PCR duplicates from the alignment files using the Picard toolkit (https://broadinstitute.github.io/picard/). Next, utilizing tools from the Genome Analysis Toolkit (GATK), base quality score recalibration (BQSR) and local realignment around known INDELs were carried out to refine the accuracy of the alignments [24]. The GATK best practices workflow was applied to identify and filter out the final genomic variants (SNPs). Haplotypes were inferred and imputed from variant calling files by using BEAGLE software (V.4.1) [25]. All discovered variations (9,764,316) were then filtered to ensure that they had at least 25 and 40 mapping and genotyping qualities, respectively. To eliminate potential genotyping errors, all discovered loci containing multiple alleles (more than 2 alleles) and inside clusters (lower than 3 SNPs in a 10 bp window) were eliminated from additional analysis [26].

### Genome-wide patterns of genomic structure and admixture

Following the removal of low-quality sequencing reads, to assess the genetic relatedness among all studied goat individuals, we used 7,843,129 SNPs retrieved from data from full genome resequencing to construct a maximum-likelihood (ML) phylogenetic tree [27]. To manage and visualize the topological structure, the free available tool (iTOL, https://itol.embl.de/) was utilized. To generate pruned SNP data with approximate linkage equilibrium (LD), all genomic data was pruned for LD in the PLINK program (“indeppairwise 50 10 0.1” flag) [28]. In addition, PCA was carried out through the use of the genome wide complex trait (GCTA) statistical approach [29]. To estimate the genomic structure of the studied goat samples, we employed the admixture software clustering process (v. 1.3.0) with an ancestor population (*K*) size ranging from *K* = 2 to *K* = 6 (10,000 times over in each run) [30]. By applying CHROMOPAINTER and fineSTRUCTURE programs, the haplotype sharing patterns were explored for all populations [31]. Additionally, by using the PLINK program [28] with default parameters, we estimated different genomic features, including LD decay, inbreeding (F) (‘-het’ flag), and nucleotide diversity.

### Genome-wide selective sweep analysis

To discover genomic selection signals related to physiological traits, two distinct approaches were applied. The genome-wide weighted *F*ST was estimated [32] in order to calculate genetic distance among populations with different sample sizes [33]. In addition, by using VCFtools (V0.1.13), we investigated nucleotide diversity θπ (-Window-pi 50 kb -Window-pi-step 25 kb) for the studied populations [34]. The sliding window analyses were carried out with a 50 kb window size and a 25 kb step size for the entire genome. The average log2 (θπ Pakistan group/θπ Northeast Asia) and *F*ST scores of SNPs in each window were computed.

### Gene set enrichment and pathway analysis

To investigate the potential pathways associated with the discovered genomic regions, using the aforementioned methods (*F*ST and log2 ratio), the Variant Effect Predictor (VEP) toolkit from Ensembl was used (https://ensembl.org/info/docs/tools/vep/index.html) to annotate all candidate genomic regions. Gene set enrichment analysis was then performed using a reliable and up-to-date tool for functional enrichment analysis (g: Profiler; https://biit.cs.ut.ee/gprofiler/). Finally, we used Benjamini-Hochberg’s False Discovery Rate (FDR-BH) adjustment across all tests to correct thresholds for false discovery rates.

## Results

### Population structure and genomic diversity

**Fig. 1:**
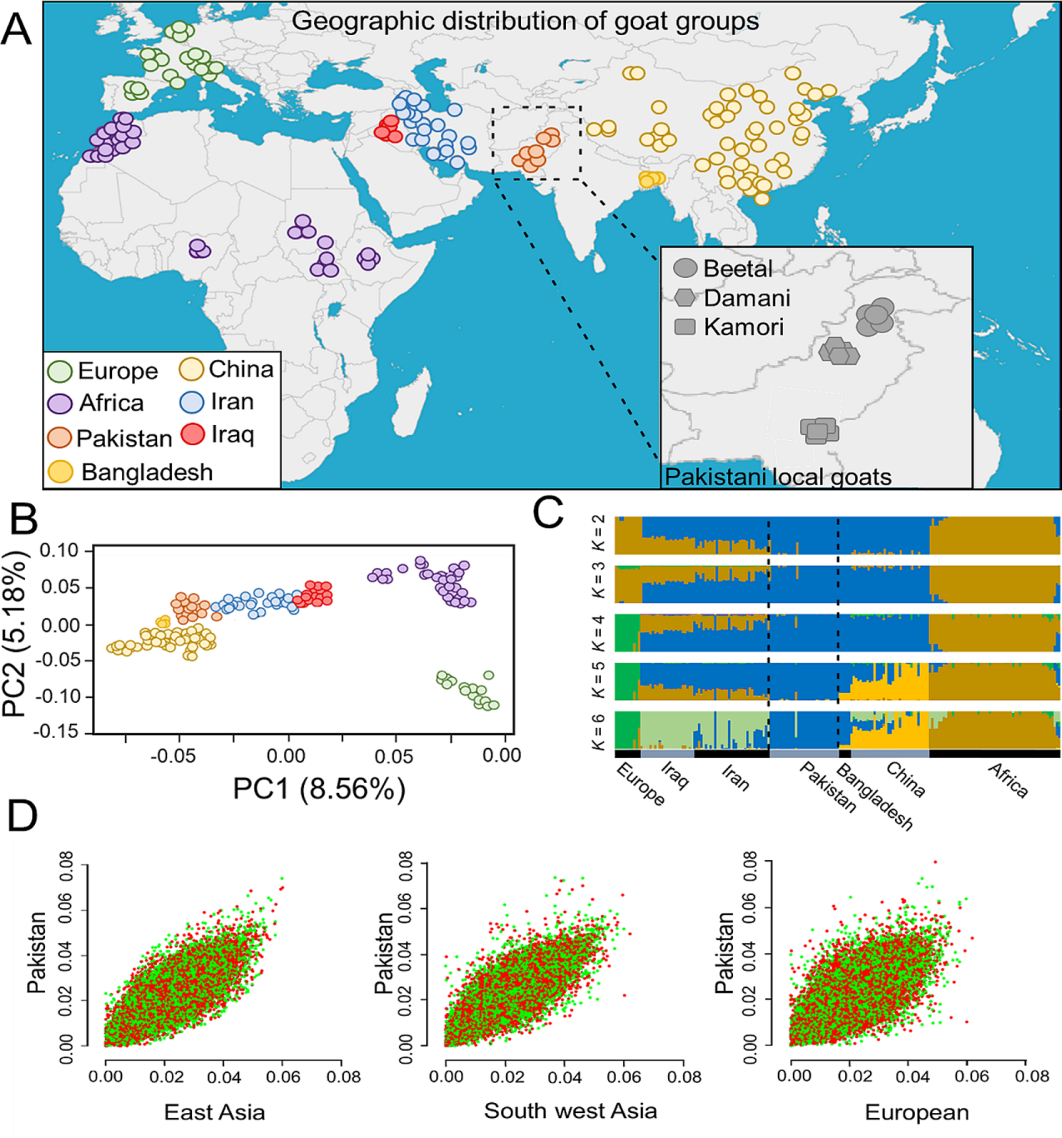
Geographic distribution of domestic goats analyzed in the current study (**A**). The color of goat populations reflects their geographic regions. (**B**) PCA analysis with whole genome data, PC1 against PC2. (**C**) ADMIXTURE clustering analysis for studied goat populations (ancestral populations from *K* = 2 to *K* = 6). (**D**) Nucleotide diversity correlations (non-overlapping window with 50-kb) between Pakistani native goat groups and other goat populations (orange)

All the studied goat individuals were assigned to seven different groups according to their geographical regions, including Europe (*n* = 20), Africa (*n* = 56), Pakistan (*n* = 26), Bangladesh (*n* = 7), China (*n* = 42), Iran (*n* = 36), and Iraq (*n* = 23) (Fig. 1 and Supplementary Table S1).

To comprehend the genetic heritage of worldwide goat populations, we conducted PCA analysis on all the studied goat individuals (Fig. 1B). The PC1 and PC2 explained 8.56% and 5.18% of the total genotypic variation, respectively. PC1 divided European and African goat groups from each other and also from Asian goat individuals. In agreement with the previous studies, those samples from East African regions showed a relatively close relationship with those from West Asian groups [2, 3]. In contrast, PC2 indicated the variation between goat samples from northern Europe and those from African regions. With whole-genome sequence data, a ML phylogenetic tree divided all samples into three main geographic subgroups, including China-Bangladesh-Pakistan; Iran-Iraq; Africa-European (Fig. S1).

By utilizing a model-based Bayesian technique that ADMIXTURE software has put into place, we explored potential admixture between all studied populations (from *K =* 2 to *K =* 6) (Fig. 1C). The *K* = 2 splits Asian goat samples from both African and European populations. At *K* = 4, with the least amount of CV error (– 0.52) (Fig. S2), we observed a division between the European and African goat populations (Fig. 1C). Ancestral genomic proportions at *K* = 5 showed that Chinese and Bangladeshi samples were mainly assigned to the same genomic clade, while Pakistani goats were divided from the remaining Asian goat groups. We next used phylogenetic tree analysis using only the examined Pakistani goats in order to concentrate on the variety within the breeds of Pakistani goats. Our results revealed that Pakistani goat individuals can be clearly grouped into three different clades (Fig. S3).

**Fig. 2:**
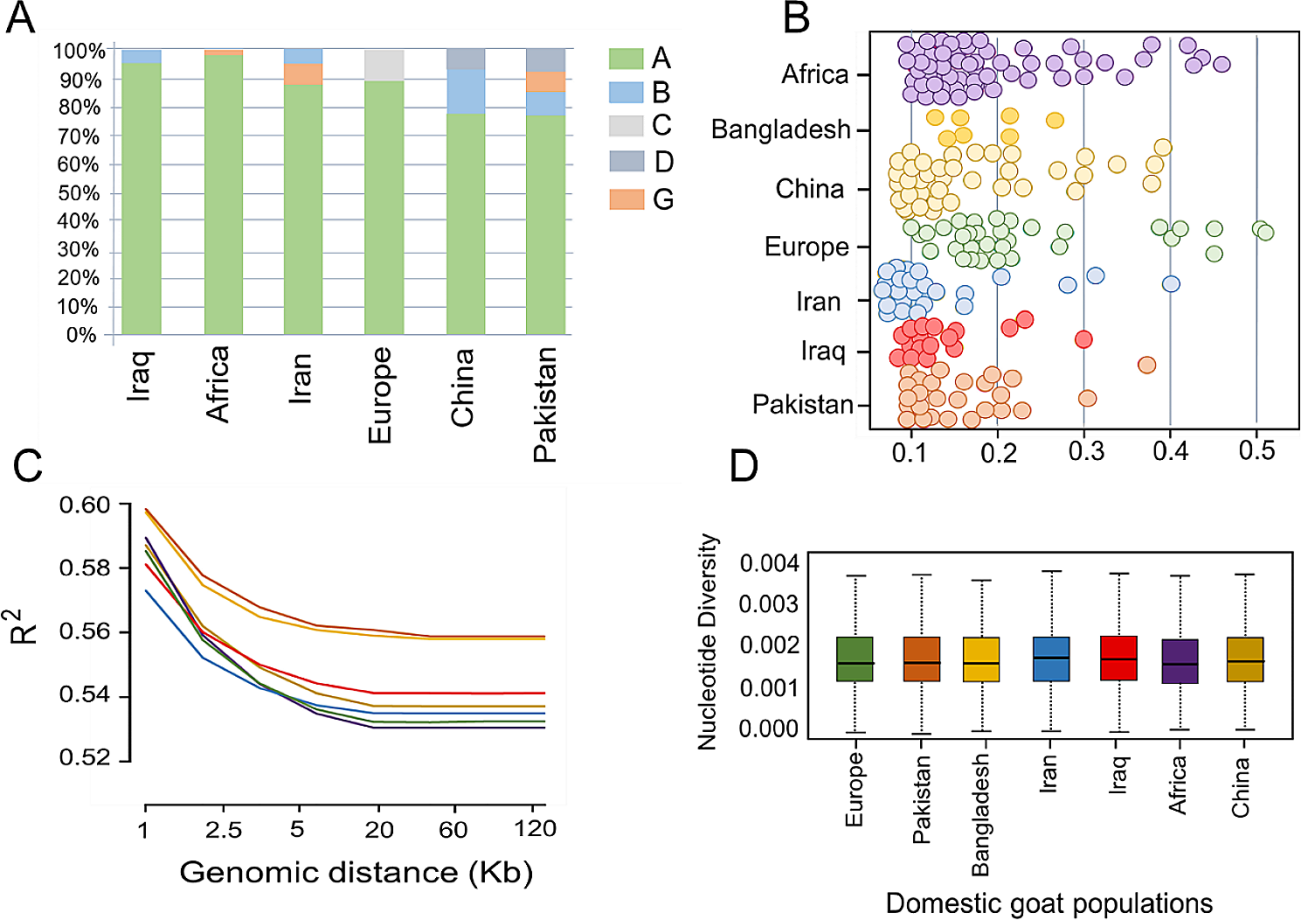
Mitochondrial genome haplotype analysis (**A**). The majority of samples fell into haplogroup A. (**B**) Inbreeding coefficient. (**C**) Linkage disequilibrium (LD) decay is calculated as the squared genomic correlation coefficient by pairwise physical distance in all domestic groups. (**D**) Nucleotide diversity across the complete genome, estimated in a sliding window of 50 KB, with increments of 20 KB

To explore the patterns of haplotype sharing between the studied goat populations, we applied the algorithms available in CHROMOPAINTER and fineSTRUCTURE (Fig. S4). The results showed a relatively higher level of haplotype sharing in Pakistani samples with East Asian goat populations, compared to other goat groups. In order to assess the historical and evolutionary processes that shape the genetic structure of local goats from Pakistan, we explore the genetic divergence patterns as a function of θπ (Fig. 1D). The results revealed a higher correlation between the gene pool of Pakistani goats and East Asian individuals (samples from China) than other populations from South west Asia (Iran and Iraq) and European (Fig. 1D). Based on the complete mitochondrial genome sequences, five different haplotypes; A, B, C, D and G were detected in the studied goat groups (Fig. 2A).

The majority of samples belong to haplogroup A (– 89%), while compared to other haplogroups, haplogroup C showed the lowest frequency (– 0.05) and was only presented in European goat individuals (Fig. 2A). The Pakistani goat individuals with four haplogroups (A, B, G and D) were the most diverse group, while the African (A and G), European (A and C) and Iraqi goat (A and B) groups harbored only two haplogroups. Genomic inbreeding coefficients were further calculated per individual (ranged from – 0.02 to – 0.54) and each goat population (ranged from 0.22 to 0.09 for European and Iranian goat populations, respectively) (Fig. 2B). We then estimated genome-wide LD (R^2^) decay rates between adjacent nucleotides across the whole sequence, which could be informative for the effective population size, non-random mating and migration events.

Our findings from the LD decay up to a distance of 120 kb are shown in Fig. 2C. At a marker pair distance of about 1 Kb, we discovered the r^2^ scores were higher for all studied goat groups (ranging from – 0.575 to – 0.592 for Iranian and Pakistani goats, respectively); however, a progressive decline was noted when the physical distance between SNPs increased up to 20 Kb. Average nucleotide diversity within each goat group was calculated, and compared to other groups, African and Iranian populations showed slightly lower and higher diversity, respectively (Fig. 2D). Our demographic analyses based on the Sequential Markov Coalescent (SMC) method reveal that the divergence times between different groups predated the estimated domestication time around 10,500 years ago (Fig. S5) [2].

### Signals of genomic positive selection associated with adaptive and milk production traits

The adaptation of domestic animals located in tropical regions to hot environmental conditions may have influenced their physical characteristics, such as hair color and skin pigmentation, which are directly associated with heat loss capacity [35]. For example, it has been reported that indigenous sheep breeds in hot and desert climates have coarse wool coats [36], while the majority of goat populations in cold geographical regions produce cashmere wool [37]. Generally, Pakistan has a tropical climate with an extreme annual range of temperature, which ranks it globally among the top ten most prone nations to climate change [38]. In order to discover candidate genes related to adaptations to local climate and milk production traits, we compared local goat breeds from Pakistan with those from East Asian samples using the *F*ST statistic and also differences in nucleotide diversity (π ln-ratio East Asian goats/local dairy goats from Pakistan).

**Fig. 3:**
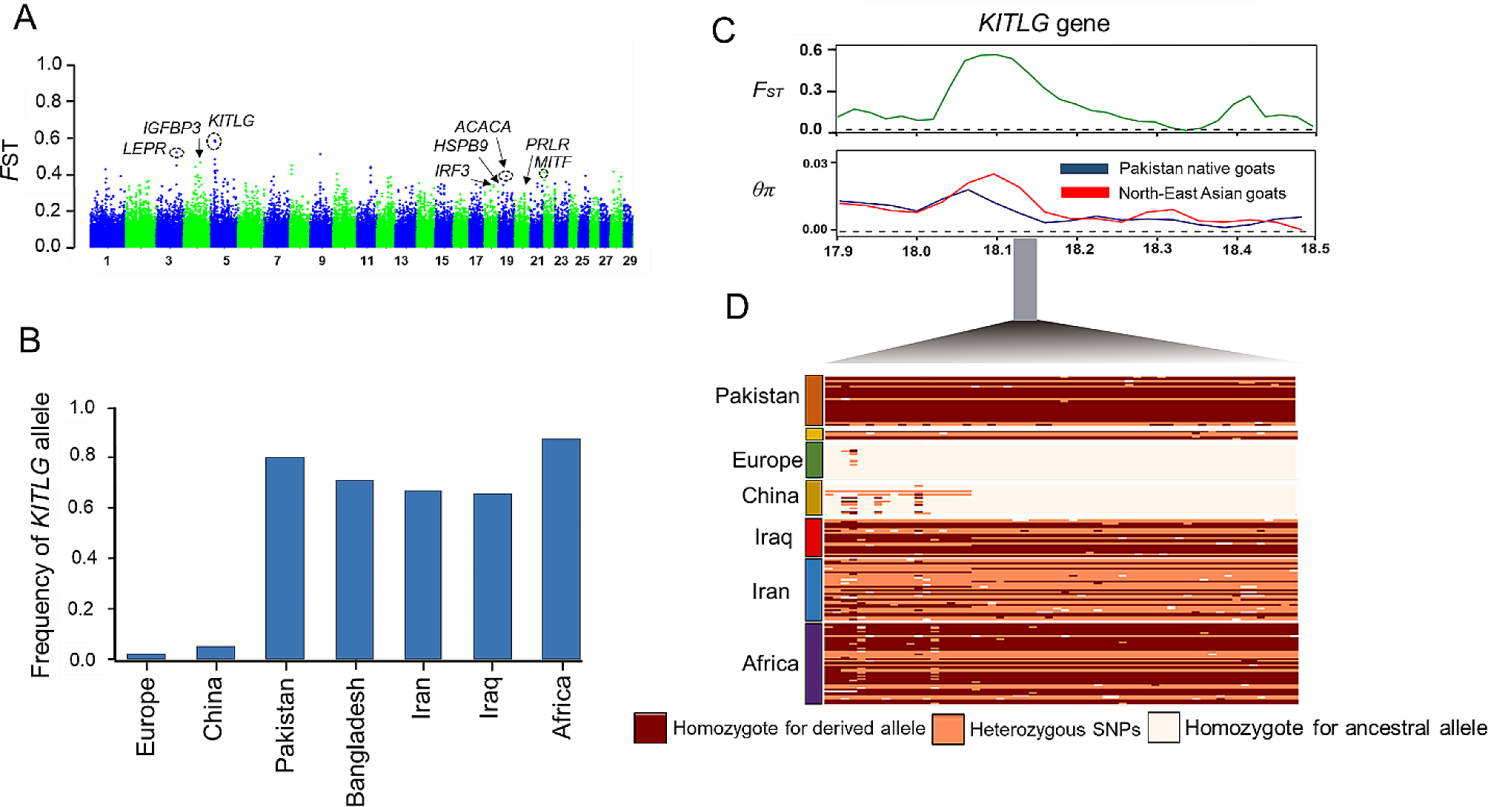
Genomic landscape of population differentiation by *F*ST between Pakistani native goats and Northeast Asian breeds (**A**). (**B**) The *KITLG* allele frequency (chromosome 5: 18, 100–18, 150 kb) in each goat population. (**C**) The putative sweep region was further validated by *F*ST and π tests. (**D**) The SNP frequency pattern in the *KITLG* gene (18, 100–18, 150 kb) is shown

Our findings from the signature of selection statistics provided a total of 145 and 147 genomic windows in the top *F*ST and log2 ratio (1% cutoff) values, respectively (Fig. 3, Tables S2 and S3). To explore the potential genetic mechanisms linked with biological traits in goats, all genomic regions were then annotated with their biological pathways (Tables S4 and S5). The strongest *F*ST-outlier signal contained one candidate gene, the *KITLG* gene, located on chromosome 5 (18, 100–18, 150 kb), that is reportedly associated with hair-related traits in humans [39] and also adaptation to local climate in livestock species [40]. We further observed that the selected genomic region had a high frequency in goat populations from Africa, South and Southwest Asia, compared with those samples from Northeast Asia (China) and Europe (Fig. 3B). The low π scores and high *F*ST values revealed a strong signature at this locus in Pakistani goat individuals (Fig. 3C). Furthermore, we identified several other genes with significant selection signals associated with milk production traits (such as; *PRLR*, *ACACA*, *LEPR*, *LPL* and *IGFBP3*) and environmental adaptation (such as; *NBEA*, *HSP70*, *HSPB9* and *CDH9*).

KEGG pathways mapping and over-representation analysis (ORA) revealed some functional categories that are significantly associated with adaptation to local climate and immune response traits including; “regulation of response to stress” (GO:0080134), “immune effector process” (GO:0002252), “regulation of immune system process” (GO:0002682). Additionally, we identified “regulation of phosphorylation” (GO:0042325), “phosphorus metabolic process” (GO:0006793) and “phosphorylation” (GO:0016310) categories that are related with milk production traits (Tables S4 and S5).

**Table 1:** Two statistical methods (*F*ST and log2 θπ ratio) were used to identify genes involving hot climate and immune response traits in Pakistani indigenous goats

Statistical-approaches	Gene	Chr.*	Position (Kb)	Summary of gene function
*F*ST (top 1%)	*KITLG*	5	18, 075–18, 125	Adaptation to hot climate [3]
	*HSPB9*	19	41, 875–41, 925	Heat stress [51]
	*DNAJB14*	6	24, 975–25, 025	Heat stress [63]
	*HSPA12B*	13	50, 800–50, 850	Heat stress [52]
	*DNAJC1*	13	22, 425–22, 475	Heat stress [64]
	*IRF3*	18	57, 025–57, 075	Immune response [54]
	*MITF*	22	31, 525–31, 575	Innate immune signaling [55]
	*RFC2*	25	32, 925– 32, 975	Immune response [56]
Log2 (θπ·Group-1 /θπ Group-2) ** (top 1%)	*KITLG*	5	18, 100–18, 150	Adaptation to hot climate [3]
	*NBEA*	12	60, 575– 60, 625	Adaptation to hot climate [65]
	*HSP70*	23	22, 400– 22, 450	Heat stress [53, 66]
	*HSPA12B*	13	22, 425–22, 475	Heat stress [52]
	*HSPB9*	19	22, 425–22, 475	Heat stress [51]
	*DNAJB14*	6	24, 975– 25, 025	Heat stress [63]
	*DNAJC1*	13	22, 425–22, 475	Heat stress [64]
	*IL23A*	5	56, 350–56, 400	Resistance to gastrointestinal parasite [57]
	*MITF*	22	31, 525– 31, 575	Immune response [55]
	*STAT2*	5	56, 350–56, 400	Immune response [67]
	*IRF3*	18	57, 025–57, 075	Immune response [54]
	*CDH9*	20	44, 550–44, 600	Environmental adaptation [68]
	*LRFN5*	21	52, 050– 52, 100	Immune response [69, 70]

* Chromosome

** Group-1 = Pakistani native goats, Group-2 = North-East Asian native goat group

## Discussion

### Genomic diversity and population structure

Knowledge about the genetic architecture and variability can advance our understanding of the evolution of a population, which could be useful for breeding and genetic conservation programs [41]. In this research, by collecting sequencing data from worldwide goat populations, we investigated the genomic structure of Pakistani native goats and their phylogenic relationship with other goat populations. Our phylogenetic analysis showed that the native goats from Pakistan are the sister taxon to samples from Bangladesh, which could be considered a separate clade from other goat breeds from Southwest Asia; including samples from Iran and Iraq countries. Through Admixture and PCA analysis, the genetic distance patterns between the analyzed goat groups were also observed. We further observed a relatively higher correlation of nucleotide diversity between Pakistani goat breeds and samples from North-East Asia, compared with samples from Southwest Asia. These results agree with those of the earlier research, which suggested that the genomic architecture of the goat populations from Southwest Asia has been affected by broad historical processes such as gene flow from ancient periods [2]. In line with previous studies on different species [3, 16, 26, 42], we discovered that all groups experienced the same pattern of reduction in LD decay as the genomic distance (Kb) increased, while a relatively rapid change in LD over increasing physical distance was detected in the African population (Fig. 2C). The r^2^ values found in this investigation ranged from – 0.53 to – 0.57 at a marker pair distance of 120 Kb, which were consistent with those previously reported for Iranian and Chinese native goat groups [16, 42]. Furthermore, the results demonstrated that Pakistani goats had much higher levels of LD across all genomic lengths than other goat groups, which could be due to recent artificial selection for economic traits in these breeds [43].

### Selection signals related to adaptation to tropical environmental and milk production traits

Due to their long history of natural selection, domestic animals are an excellent model for studying the genetic variants enabling adaptation to local climates and also for biomedical research [44]. Generally, due to their fast metabolic rate and growth performance, farm animals are susceptible to heat stress [45]. Prior research has demonstrated that heat tolerance is one of the most demanding challenges for domestic animals that inhabit tropical regions, which can negatively impact their health and production [46, 47]. In several independent studies, it has been confirmed that there is variation in the performance of goats from different breeds under high temperature conditions [48]. Thus, genetic selection could be a cost-effective tool to help improve the thermotolerance of animals in hot regions. Therefore, identifying and selecting genes related to heat tolerance is expected to provide a potential and long-term solution to this issue. In this study, in order to understand how native goats from Pakistan have adapted to the tropical climates, we compared them with samples from Northeast Asia to uncover loci that have been exposed to long-term natural selection. Our findings from the detection of selection signals revealed that some candidate loci inside the regions of high confidence selection (highest 1% log2 θπ ratio and 1% *F*ST values) may be related to adaptation to local climate and immune response processes (Table 1). We observed the strongest genomic signal (both θπ ratio and *F*ST methods) related to hair and skin pigmentation (*KITLG*) located on chromosome 5 [3, 39]. The protein related to this gene, the tyrosine kinase protein KIT, is required for the growth and differentiation of various cell types, such as melanocyte proliferation, pigment production, blood cells, and also germ cells [49]. A previous study on the human genome has reported a causative base-pair change at the enhancer of this gene (encoding the *KIT* ligand), which controls the expression of hair follicles [39]. So far, various mutations of the *KITLG* gene related to coat color and environmental adaptation [39, 40, 50] have been reported. Due to the fact that the frequency of the *KITLG* allele was higher in populations residing in hot climate conditions (such as in South Asia and Africa), we speculate that this gene may have played a role in the evolution of goat lineages (Fig. 3D). Aside from this gene, several genes for heat shock protein (HSP) were also discovered, such as; *HSPB9*, *HSP70* and *HSPA12B*, may have an impact on attributes related to heat tolerance in goats and different livestock species [51–53] (Table 1). We further discovered a few potential loci on different chromosomes that could be important in the goat immune system. For instance, we found *IL23A*, *IRF3, RFC2* and *MITF* genes that are related to immune response traits [54–57].

Due to the fact that studied Pakistani native goats are considered to be among the highest milk yielding breeds in Pakistan [12–14], comparative genome analysis of this group with other native breeds that usually produce low levels of milk production is a promising method for determining genetic diversity in milk yield characteristics. To accomplish this goal, we employed two statistical techniques: comparisons between two groups and comparisons within a group. Within the genomic regions with greater *F*ST scores (top 0.01) and reduced nucleotide diversity (cutoff 1% log2 θπ ratio), we detected some candidate genes associated with milk production traits. Protein-encoding genes were listed in Table 2.

**Table 2:** Identified candidate genes related with milk production-related traits

Statistical-approaches	Gene	Chr.*	Position (Kb)	Summary of gene function
*F*ST (top 1%)	*LEPR*	3	41,100 − 41,150	Milk fat and protein [71, 72]
	*LPL*	8	66,725 − 66,775	Milk fat yield [16, 62]
	*IGFBP3*	4	44,100 − 44,150	Milk performance [73]
	*TRNAC-ACA*	6	66,425 − 66,475	Milk composition traits [74]
	*TRNAS-GGA*	4	61,925 − 61,975	Milk composition traits [74]
	*PRLR*	20	38,950 − 39,000	Milk production traits [75, 76]
	*ABCB1*	4	87,525 − 87,575	Milk production traits [77]
	*ACACA*	19	13,325 − 13,375	Milk fat content [78, 79]
	*TSHR*	10	10,650 − 10,700	Milk production [80]
	*SUPT3H*	23	30,575 − 30,625	Milk protein percentage [81]
Log2 (θπ·Group-1 /θπ Group-2) ** (top 1%)	*ARFGEF1*	14	50,700 − 50,750	Milk production [82, 83]
	*TRNAC-ACA*	5	16,725 − 16,775	Milk composition traits [74]
	*TRNAS-GGA*	4	12,125 − 12,175	Milk composition traits [74]
	*LEPR*	3	41,100 − 41,150	Milk fat and protein [71, 72]
	*LPL*	8	66,750 − 66,800 and 66,725 − 66,775	Milk fat yield [16, 62]
	*IGFBP3*	4	44,125 − 44,175 and 44,100 − 44,150	Milk performance [73]
	*TSHR*	10	10,625 − 10,675	Milk production [80]
	*AGPAT1*	23	22,700 − 22,750	Milk fatty acid [84, 85]
	*SUPT3H*	23	30,500 − 30,550	Milk protein percentage [82]

* Chromosome

** Group-1 = Pakistani native goats, Group-2 = North-East Asian native goat group

For example; in the cluster of signals discovered by both statistical methods, we found *IGFBP3* as a putative candidate gene on chromosome 4 (44.10-44.15 MB) (Table 2). This gene encodes a protein with an IGFBP domain, which is a subfamily of the insulin-like growth factor binding protein [58]. It has been confirmed that the mutations in this gene are related to the milk production traits in both goat [59] and sheep [60] species. Another gene associated with milk, *LPL*, was found in one of the selection regions of chromosome 8, which is a crucial potential gene that plays a central role in human plasma triglyceride metabolism [61]. Previous studies have reported that genetic mutations in the *LPL* gene are related to milk production traits (e.g., milk yield, fat content, and protein yield) and composition traits (protein and fat percentages) in dairy goats and also in different livestock species [16, 62].

## Conclusions

By exploring genome-wide data from worldwide goat populations, we provided valuable insight into the genomic architecture of three local goat populations in Pakistan. The results indicate that there is a relatively higher genetic affinity between studied goat samples from Pakistan and those from North-East Asia, compared with samples from other geographical regions. We further observed high levels of LD decay in Pakistani goat samples, which may be a consequence of recent human selection for interested traits (such as milk production traits). Furthermore, we discovered multiple candidate genes related to local climate adaptation, immune response, and milk production traits. The genomic regions discovered in this study will help us better understand the mechanisms of selection and identify the targets of selection in goat breeds located in tropical regions.

### Electronic supplementary material

Below is the link to the electronic supplementary material.

Supplementary Material 1

Supplementary Material 2

## Data Availability

The data used to support the findings of this study have been archived at the NCBI SRA Database under the BioProject accession number PRJNA1087734. The fastq files used in this study were downloaded from the public sequence database (https://trace.ncbi.nlm.nih.gov/Traces/sra).

## Abbreviations

BAM: Binary Alignment MAP
GATK: Genome Analysis Toolkit
BWA: Burrows-Wheeler Aligner
LD: Linkage disequilibrium
FST: Fixation index
GO: Gene ontology
PCA: Principle component analysis
SNP: Single-nucleotide polymorphism
ROH: Runs of homozygosity
