# Supplementary material for: Genomic analysis uncovers novel candidate genes related to adaptation to tropical climates and milk production traits in native goats
Source: BMC Genomics. 2024 May 14;25:477. doi: 10.1186/s12864-024-10387-y (PMC11094986; doi:10.1186/s12864-024-10387-y)

**Genomic analysis uncovers novel candidate genes related to adaptation to tropical climates and milk production traits in native goats**

Chenxi Zhang ^1^, Hojjat Asadollahpour Nanaei ^1,2^*, Niloufar Jafarpour Negari^3^, Mahmoud Amiri Roudbar ^4^, Zeinab Amiri Ghanatsaman^2^, Xiaojun Yang ^1^*

^1^College of Animal Science and Technology, Northwest A&F University, Yangling, Shaanxi 712100, China

^2^ Animal Science Research Department, Fars Agricultural and Natural Resources Research and Education Center, Agricultural Research, Education and Extension Organization (AREEO), Shiraz, Iran

^3^ Department of Animal Science, Shahid Bahonar University of Kerman, Kerman 7616914111, Iran

^4^ Department of Animal Science, Safiabad-Dezful Agricultural and Natural Resources Research and Education Center, Agricultural Research, Education and Extension Organization (AREEO), Dezful 333, Iran

*Corresponding Authors:

Xiaojun Yang : [yangxj@nwafu.edu.cn](mailto:yangxj@nwafu.edu.cn)

Hojjat Asadollahpour Nanaie: [h.asadollahpour@agr.uk.ac.ir](mailto:h.asadollahpour@agr.uk.ac.ir)

**Fig. S1**

ML phylogenetic tree of 210 goat individuals from different geographical regions. Branch color shows membership in different geographical populations.


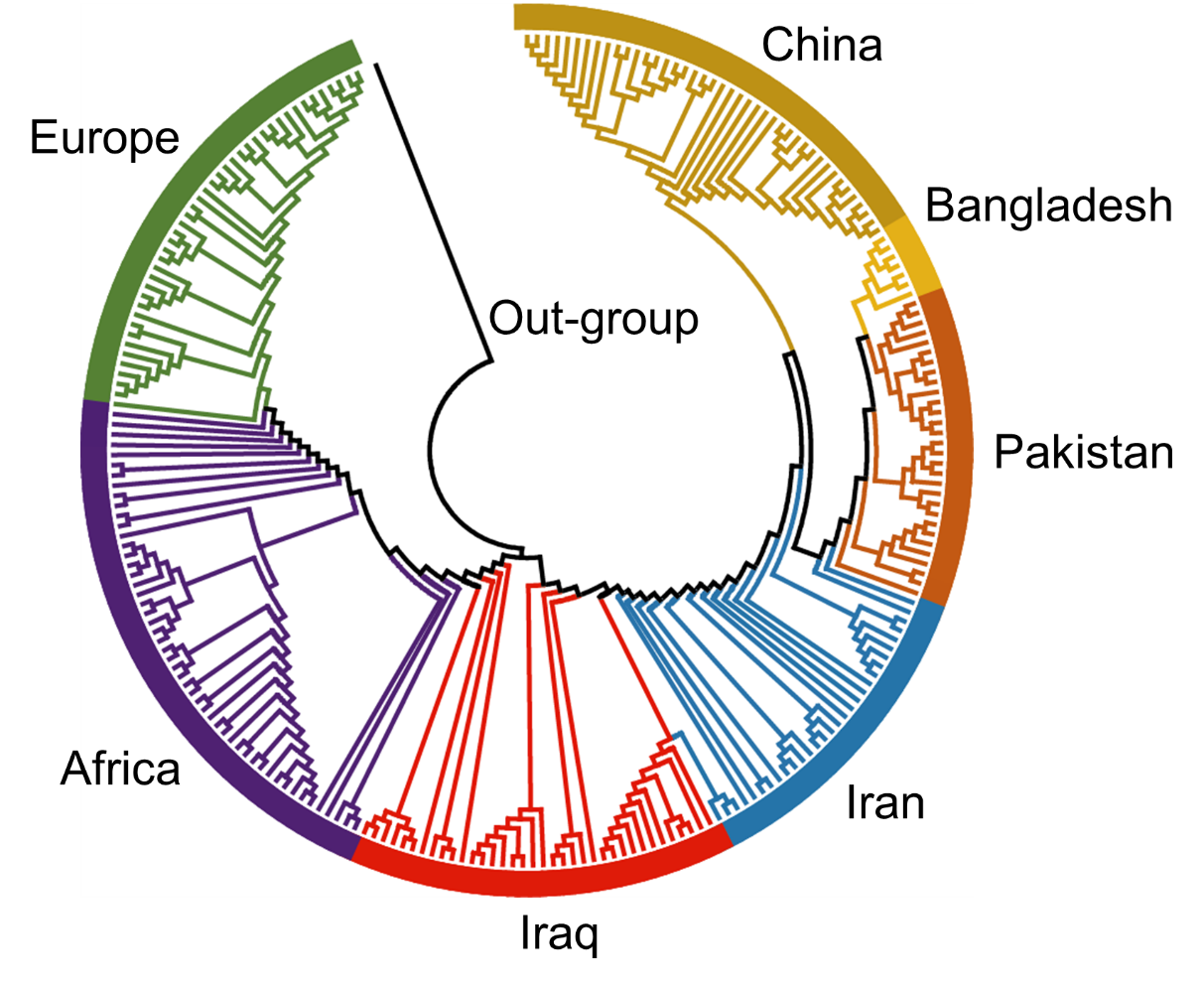


**Fig. S2**

Cross validation error (CV) plot from ADMIXTURE. At K = 4, with the lowest CV error


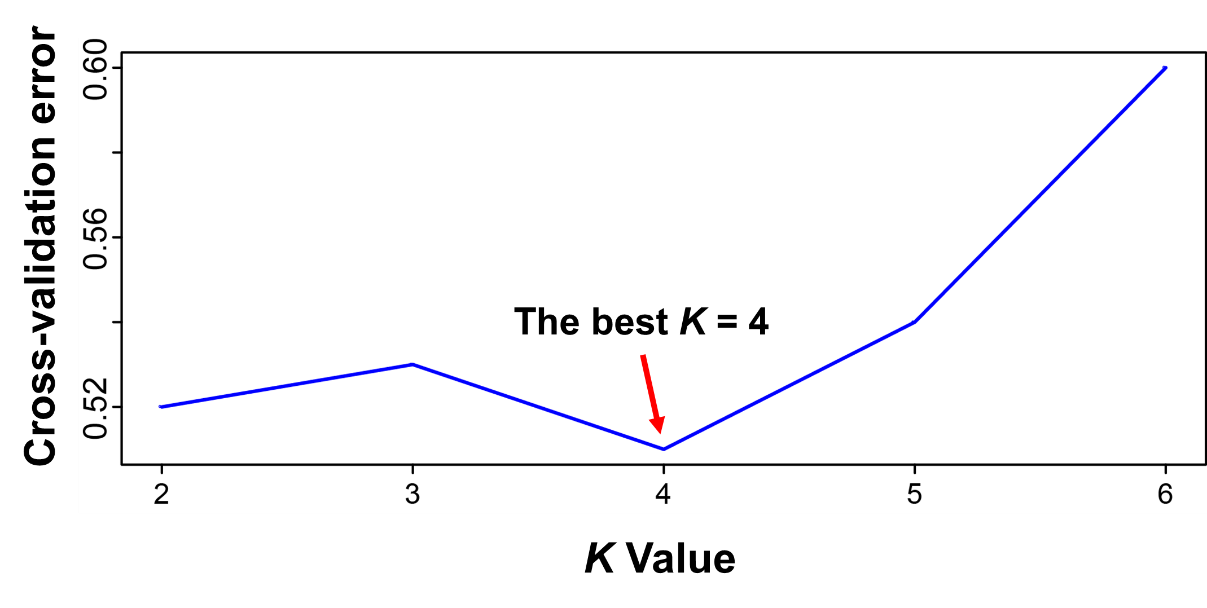


**Fig. S3**

Phylogenetic tree of Pakistani goats constructed using Neighbor-Joining method.


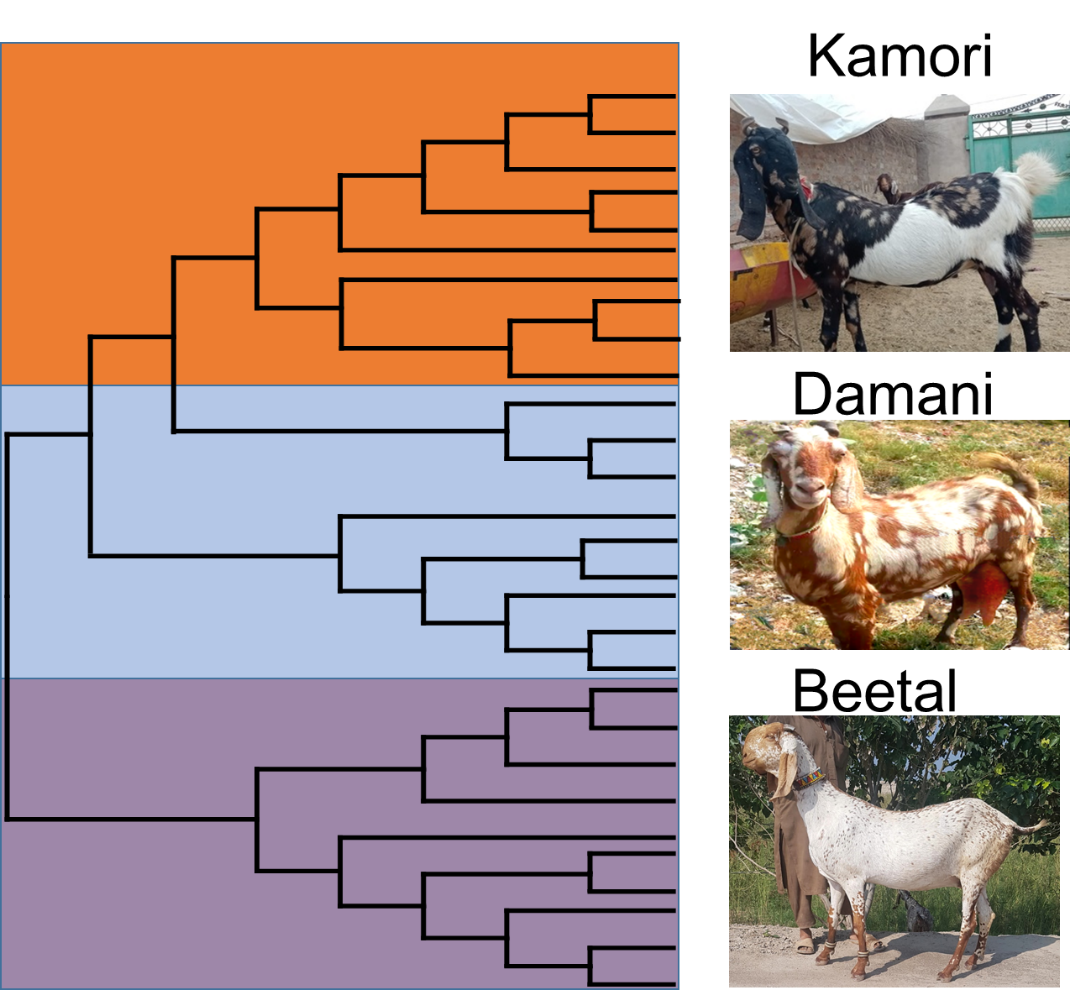


**Fig. S4**

Population relationships from co-ancestry matrix calculated by ChromoPainter/fineSTRUCTURE. Each column shows the donor individuals and row corresponds to the recipient genomes. Pakistani goats showed relatively higher haplotype sharing with samples from east Asia (red box), compared with other Asian goat populations.


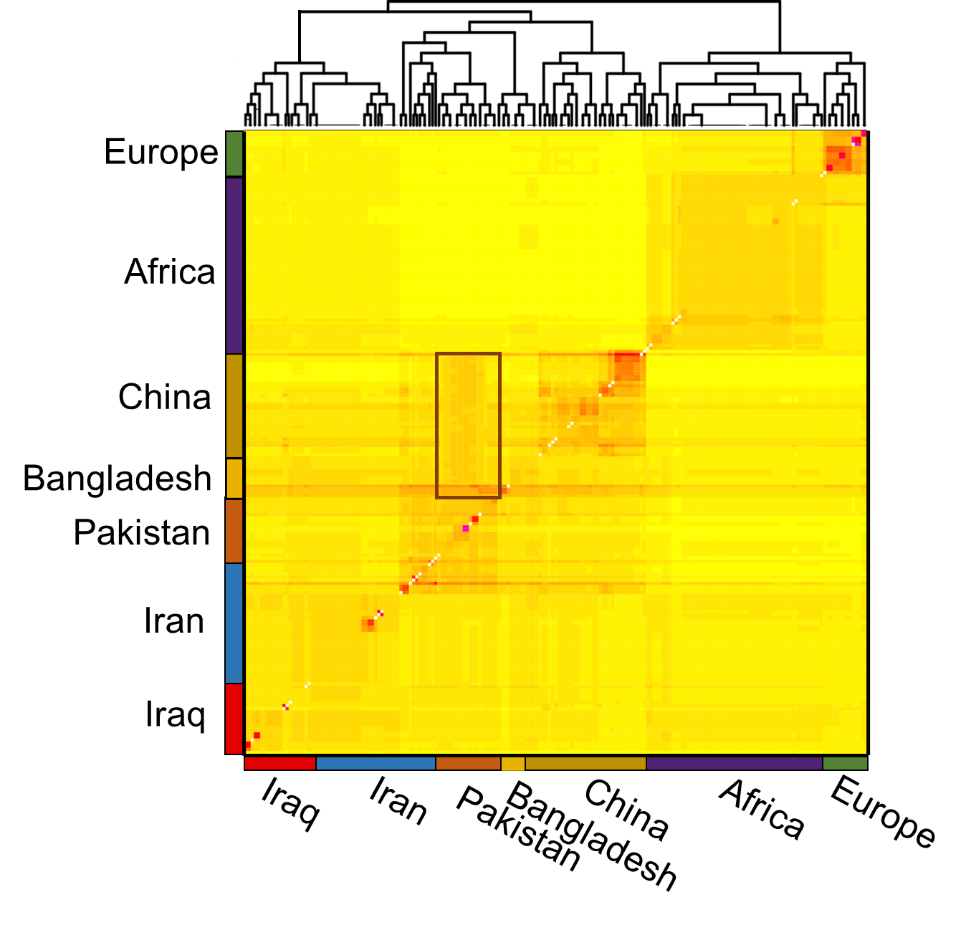


**Fig. S5**

Inference of population size from whole-genome sequences. Effective population size histories inferred using SMC++ for all studied groups (with 20 bootstrap replicates).


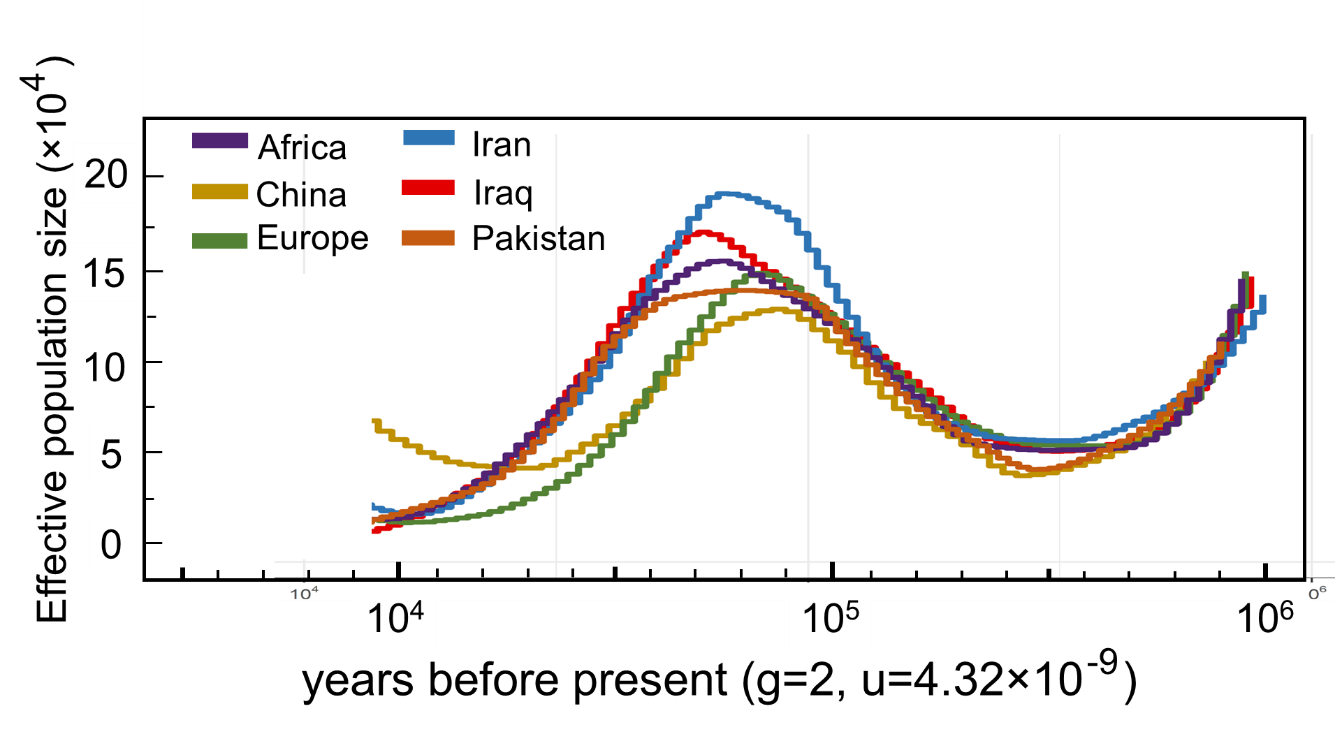

Supplement: Supplementary file 2 — Supplementary Material 2 [file 12864_2024_10387_MOESM2_ESM.docx]
